# Supplementary material for: Multi‐dataset identification of innovative feature genes and molecular mechanisms in keratoconus
Source: J Cell Mol Med. 2024 Sep 19;28(18):e70079. doi: 10.1111/jcmm.70079 (PMC11412914; doi:10.1111/jcmm.70079)
Supplement: Supplementary file 2 — Material S2. [file JCMM-28-e70079-s002.zip › 7.GSEA analysis/1.ARL11/KEGG_STARCH_AND_SUCROSE_METABOLISM.html]

Details for gene set KEGG\_STARCH\_AND\_SUCROSE\_METABOLISM[GSEA]

|  || Dataset | ARL11.group.cls#h\_versus\_l |
| Phenotype | group.cls#h\_versus\_l |
| Upregulated in class | h |
| GeneSet | KEGG\_STARCH\_AND\_SUCROSE\_METABOLISM |
| Enrichment Score (ES) | 0.47087857 |
| Normalized Enrichment Score (NES) | 1.4422722 |
| Nominal p-value | 0.08301158 |
| FDR q-value | 0.96900773 |
| FWER p-Value | 0.848 |
Table: GSEA Results Summary

  

Fig 1: Enrichment plot: KEGG\_STARCH\_AND\_SUCROSE\_METABOLISM      
 Profile of the Running ES Score & Positions of GeneSet Members on the Rank Ordered List

  

| SYMBOL | TITLE | RANK IN GENE LIST | RANK METRIC SCORE | RUNNING ES | CORE ENRICHMENT || 1 | GBA3 | na | 822 | 0.413 | 0.0648 | Yes |
| 2 | HK3 | na | 938 | 0.397 | 0.1465 | Yes |
| 3 | UGT2B7 | na | 1178 | 0.368 | 0.2184 | Yes |
| 4 | PYGM | na | 1375 | 0.350 | 0.2877 | Yes |
| 5 | UGT2B15 | na | 2914 | 0.244 | 0.2957 | Yes |
| 6 | UGT2B11 | na | 2923 | 0.244 | 0.3478 | Yes |
| 7 | UGT2B4 | na | 3576 | 0.216 | 0.3753 | Yes |
| 8 | UGT1A8 | na | 3762 | 0.209 | 0.4148 | Yes |
| 9 | SI | na | 3937 | 0.203 | 0.4532 | Yes |
| 10 | UGT1A6 | na | 4803 | 0.172 | 0.4650 | Yes |
| 11 | UGT2B28 | na | 5683 | 0.146 | 0.4709 | Yes |
| 12 | GYS2 | na | 7764 | 0.097 | 0.4317 | No |
| 13 | PYGB | na | 9443 | 0.068 | 0.3979 | No |
| 14 | UGT2B10 | na | 9893 | 0.062 | 0.3981 | No |
| 15 | GANC | na | 9905 | 0.061 | 0.4110 | No |
| 16 | GYS1 | na | 11583 | 0.041 | 0.3713 | No |
| 17 | ENPP3 | na | 12321 | 0.033 | 0.3572 | No |
| 18 | AMY1C | na | 12650 | 0.030 | 0.3541 | No |
| 19 | UGT2A3 | na | 13634 | 0.022 | 0.3305 | No |
| 20 | GAA | na | 13707 | 0.021 | 0.3329 | No |
| 21 | AGL | na | 13809 | 0.020 | 0.3344 | No |
| 22 | GBE1 | na | 13827 | 0.020 | 0.3382 | No |
| 23 | GUSB | na | 16664 | -0.001 | 0.2567 | No |
| 24 | PGM2 | na | 17963 | -0.010 | 0.2214 | No |
| 25 | UGDH | na | 19859 | -0.023 | 0.1717 | No |
| 26 | HK1 | na | 19947 | -0.023 | 0.1742 | No |
| 27 | PYGL | na | 20281 | -0.026 | 0.1701 | No |
| 28 | UGT2A1 | na | 20689 | -0.029 | 0.1645 | No |
| 29 | UGP2 | na | 20718 | -0.029 | 0.1700 | No |
| 30 | UXS1 | na | 21580 | -0.036 | 0.1528 | No |
| 31 | GPI | na | 23113 | -0.051 | 0.1197 | No |
| 32 | TREH | na | 23509 | -0.056 | 0.1202 | No |
| 33 | ENPP1 | na | 23556 | -0.057 | 0.1310 | No |
| 34 | PGM1 | na | 25044 | -0.077 | 0.1045 | No |
| 35 | GCK | na | 25358 | -0.081 | 0.1128 | No |
| 36 | G6PC2 | na | 25991 | -0.090 | 0.1139 | No |
| 37 | PGM2L1 | na | 26041 | -0.091 | 0.1319 | No |
| 38 | HK2 | na | 26843 | -0.105 | 0.1314 | No |
| 39 | MGAM | na | 34174 | -0.448 | 0.0160 | No |
Table: GSEA details [plain text format]

  

Fig 2: KEGG\_STARCH\_AND\_SUCROSE\_METABOLISM      
 Blue-Pink O' Gram in the Space of the Analyzed GeneSet

  

Fig 3: KEGG\_STARCH\_AND\_SUCROSE\_METABOLISM: Random ES distribution      
 Gene set null distribution of ES for **KEGG\_STARCH\_AND\_SUCROSE\_METABOLISM**
